# Adverse events among Ontario home care clients associated with emergency room visit or hospitalization: a retrospective cohort study

**DOI:** 10.1186/1472-6963-13-227

**Published:** 2013-06-22

**Authors:** Diane M Doran, John P Hirdes, Regis Blais, G Ross Baker, Jeff W Poss, Xiaoqiang Li, Donna Dill, Andrea Gruneir, George Heckman, Hélène Lacroix, Lori Mitchell, Maeve O’Beirne, Nancy White, Lisa Droppo, Andrea D Foebel, Gan Qian, Sang-Myong Nahm, Odilia Yim, Corrine McIsaac, Micaela Jantzi

**Affiliations:** 1 Lawrence S. Bloomberg Faculty of Nursing, University of Toronto, 155 College Street, Suite 130, Toronto, ON, M5T 1P8, Canada; 2 School of Public Health and Health Systems, Faculty of Applied Health Sciences, University of Waterloo, Waterloo, 200 University Avenue West, Waterloo, ON, N2L 3G1, Canada; 3 Department of Health Administration, University of Montreal, Station Centre-ville, PO Box 6128, Montréal, Québec, H3C 3J7, Canada; 4 Institute of Health Policy, Management, and Evaluation, Faculty of Medicine, University of Toronto, 155 College Street, Toronto, ON, M5T 1P8, Canada; 5 Department of Health and Wellness, Nova Scotia Department of Health and Wellness, Barrington Tower, 7th Fl., 1894 Barrington Street, Halifax, NS, B3J 2A8, Canada; 6 Women’s College Research Institute, Women’s College Hospital, 790 Bay Street, 7th floor, Toronto, ON, M5G 1N8, Canada; 7 School of Public Health and Health Systems, and Research Institute for Aging, University of Waterloo, 200 University Avenue West, Waterloo, ON, N2L 3G1, Canada; 8 Saint Elizabeth Health Care, 90 Allstate Parkway, Suite 300, Markham, ON, L3R 9Z9, Canada; 9 Home Care Program, Winnipeg Regional Health Authority, 3rd Floor – 496 Hargrave Sreet, Winnipeg, MB, R3A 0X7, Canada; 10 Department of Family Medicine & Department of Community Health Sciences, University of Calgary, G-012 HSC, 3330 Hospital Drive Northwest, Calgary, AB, T2N 4N1, Canada; 11 Canadian Institute for Health Information (retired), 13 Graham Avenue, Ottawa, ON, K1S 0B6, Canada; 12 Ontario Association of Community Care Access Centres, 130 Bloor Street West, Suite 200, Toronto, ON, M5S 1N5, Canada; 13 Cape Breton University, 1250 Grand Lake Road Sydney, P.O. Box 5300, Nova Scotia, B1P 6L2, Canada

## Abstract

**Background:**

Home care (HC) is a critical component of the ongoing restructuring of healthcare in Canada. It impacts three dimensions of healthcare delivery: primary healthcare, chronic disease management, and aging at home strategies. The purpose of our study is to investigate a significant safety dimension of HC, the occurrence of adverse events and their related outcomes. The study reports on the incidence of HC adverse events, the magnitude of the events, the types of events that occur, and the consequences experienced by HC clients in the province of Ontario.

**Methods:**

A retrospective cohort design was used, utilizing comprehensive secondary databases available for Ontario HC clients from the years 2008 and 2009. The data were derived from the Canadian Home Care Reporting System, the Hospital Discharge Abstract Database, the National Ambulatory Care Reporting System, the Ontario Mental Health Reporting System, and the Continuing Care Reporting System. Descriptive analysis was used to identify the type and frequency of the adverse events recorded and the consequences of the events. Logistic regression analysis was used to examine the association between the events and their consequences.

**Results:**

The study found that the incident rate for adverse events for the HC clients included in the cohort was 13%. The most frequent adverse events identified in the databases were injurious falls, injuries from other than a fall, and medication-related incidents. With respect to outcomes, we determined that an injurious fall was associated with a significant increase in the odds of a client requiring long-term-care facility admission and of client death. We further determined that three types of events, delirium, sepsis, and medication-related incidents were associated directly with an increase in the odds of client death.

**Conclusions:**

Our study concludes that 13% of clients in homecare experience an adverse event annually. We also determined that an injurious fall was the most frequent of the adverse events and was associated with increased admission to long-term care or death. We recommend the use of tools that are presently available in Canada, such as the Resident Assessment Instrument and its Clinical Assessment Protocols, for assessing and mitigating the risk of an adverse event occurring.

## Background

The occurrence of an adverse event is a patient safety issue that has been well documented with respect to patients in acute-care settings [1]; however, there are only limited data available about safety problems experienced by clients in HC settings [2,3]. Our study was initiated to address that gap in knowledge.

Home care differs from the care provided in acute and hospital settings in four significant ways: i) the nature of the formal service provision; ii) the role of family members; iii) the characteristics of the clients/patients receiving care; and iv) the location where care is provided [2]. For example, family members often act as informal caregivers to supplement the time and contact between a HC client and HC staff. Consequently, patient outcomes are influenced not only by formal healthcare providers, but also by the quality of care provided by unpaid caregivers, and by decisions made by clients and their caregivers in their homes [4].

Home care is a care option that is increasing in practice and correspondingly in cost. The Canadian Home Care Association estimates that 1.4 million Canadians received publicly funded HC services in 2011. That is an increase of 55% since 2008 [5]. The cost of providing that care is estimated at $5.8 billion [6]. One of the reasons for this increase is the discharge from acute-care settings of patients who require continuing care. Approximately 73.4% of HC clients are reported to have been discharged from an acute-care setting [7]. With the growth in homecare comes the challenge of understanding and managing the safety issues that pertain. Those issues are only beginning to be addressed in healthcare literature; however, it is imperative that they are better understood in order to effectively develop policy and practice recommendations to address them.

In 2010 a scoping review of adverse events experienced by HC clients reported overall rates of 3.5 to 15.1% [8]. Adverse drug events, infections, wounds, and falls were the types of events identified. Policy suggestions from that review addressed the need for improved assessment, better monitoring, education strategies, and improved coordination and communication between partners in the provision of care [8]. In Canada, one of the first HC patient safety studies reported a 5.5% incidence rate of harmful incidents and/or adverse outcomes in a sample of 279 Winnipeg HC clients. That study determined that injurious falls accounted for nearly half (46%) of the referenced events [3]. Two subsequent studies, one conducted in the United States [4] and one in Canada [2], reported that 13% of HC clients experienced a harmful patient-safety incident and/or adverse outcome. The types of outcomes identified in those studies included: urinary tract infections, wound deterioration, unexpected nursing-home admission, an increase in the number of pressure ulcers, side effects of improper medication administration, hypo/hyperglycemia, and unexpected death. The studies reported that clients who experienced such events were generally older, had more depressive symptoms and behavioural problems, were more functionally impaired [2,4], had Parkinson’s disease, received psychotropic medications or were left alone for short or long periods of time [2]. Both of the studies were limited in sample size. Doran et al. addressed that limitation in a study that involved 238,958 HC clients from Ontario, Nova Scotia, and the Winnipeg Regional Health Authority [9]. That study investigated the prevalence of safety-related problems among Canadian HC clients using data collected through the interRAI Resident Assessment Instrument – Home Care (RAI-HC©). The study identified the most common adverse events and outcomes as: new falls (11%), unintended weight loss (9%), new emergency department (ED) visits (7%), and new hospital visits (8%). In the Doran et al. study the data available referenced only those HC clients who qualified for a RAI-HC assessment. Because of that limitation the findings may not be representative of all HC clients. Further, that study was not able to identify, for every incident, the adverse event that resulted specifically in an ED visit or a hospitalization. Both of these limitations have been addressed in our current study through access to the more comprehensive and integrated records that are available in Ontario.

The purpose of our study was to investigate the incidence, magnitude, and types of adverse events and subsequent outcomes experienced by the Ontario HC population. We focused on two specific outcomes of adverse events, placement in a long-term-care (LTC) facility and client death. This study was conducted in conjunction with the Pan-Canadian Home Care Safety Study [10].

### Study questions

The study questions included the following.

1. What is the incidence, among Ontario HC clients, of adverse events/outcomes that are associated with emergency room visits or hospitalization;

2. What are the most frequent types of adverse events experienced by HC clients;

3. What is the relationship between adverse events and LTC admission or death?

## Methods

The conceptualization of the patient-safety variables was guided by the World Health Organization [11] framework for safe healthcare. Its definitions were adapted to the HC context. The WHO defines patient safety as “freedom, for a patient, from unnecessary harm or potential harm associated with healthcare” (p. 7). In the context of homecare there is a unique challenge because the care may be provided by a variety of contributors: healthcare professionals, informal caregivers including family, and the clients themselves. Consequently it is often very difficult to isolate the contribution of any one of that mix of providers and thus to relate the adverse events to specific healthcare delivery.

An *Adverse event* (AE) is defined by the WHO as an injury caused by medical management or complication rather than by the underlying disease itself, and one that results in either prolonged healthcare, or disability at the time of discharge from care, or both. An *adverse outcome* is defined as consequence of an adverse event and generally includes prolonged healthcare, a resulting disability, or death at the time of discharge [11]. An adverse outcome may be partially or totally attributable to the care provided. In homecare it is often difficult to determine that causal relationship because much of the care provided is unobserved. In order to minimize the threat of detection bias when we examined adverse events we developed specific rules and inclusion/exclusion criteria for each event (Additional file 1). For example, in the case of a catheter associated urinary tract infection (UTI) the criteria included the following: ‘the UTI was recorded on any ED visit, or as a pre-admit condition for an overnight hospitalization, within 30 days after RAI-HC assessment in which an indwelling urinary catheter was documented and without UTI recorded at the time of RAI-HC assessment.’

### Study design, setting, and cohort

A retrospective cohort design was used to estimate the incidence and types of adverse events that were associated with an ED visit or hospitalization. The setting was HC services in Ontario, the largest province in Canada with a population of 13.5 million, approximately two million of which are over the age of 65 years. Healthcare is publicly funded through a universal insurance program which covers home care, both short-stay (for post-acute or rehabilitation services) and long-stay clients. Ontario has developed the kind of extensive network of health services data that we required to meet the goals of our study.

The cohort consisted of the population of HC clients who received publicly funded HC services from the province between January 1, 2008 and December 31, 2009. All reasons for HC services were included and all patients aged 18 or older at the time of service were included, with the exception of palliative clients, because of differences in their expected clinical course. The health-services utilization for acute hospital care, ED visits, long-term care, and inpatient psychiatric care by the HC population was used to identify the occurrence of adverse events.

### Ethics and data access

The study received ethics approval from the University of Toronto Research Ethics Review Board. The HC population was identified from the episode information of Canada’s Home Care Reporting System (HCRS) data. The HCRS consists of three parts: episode information (e.g. case open date, discharge date, client region), RAI-HC assessment (for long-stay clients), and health service utilization data (e.g., the number of scheduled visits and time of service). The RAI-HC© assessment instrument provides a comprehensive profile of Canadian HC clients, their environment, services and outcomes [12]. Assessments are completed on a periodic basis, including at admission for clients expected to be on service for 60 days or longer, and then bi-annually. A description of the RAI-HC and its psychometric properties can be found in Morris et al. [13] and Landy et al. [14].

The occurrence of adverse events was identified from the available databases. The pre-admit conditions for acute hospital admission were obtained from the Discharge Abstract Database (DAD) and ED visits were identified from the National Ambulatory Care Reporting System (NACRS). The pre-admit conditions for inpatient mental-health service utilization were obtained from the Mental Health Reporting System (MHRS). ICD-10 codes in NACRS/DAD data, RAI-Mental Health (MH) assessment items, and DSM-IV provisional diagnostic categories in MHRS data were used to identify adverse events. Only pre-admit diagnoses were considered, with the exception of suicide, for which both pre- and post-admit diagnoses were considered because of small numbers. Only unplanned ED visits were considered. Admission to long-term care facilities/nursing homes were reported in the Continuing Care Reporting System (CCRS).

**Figure 1:**
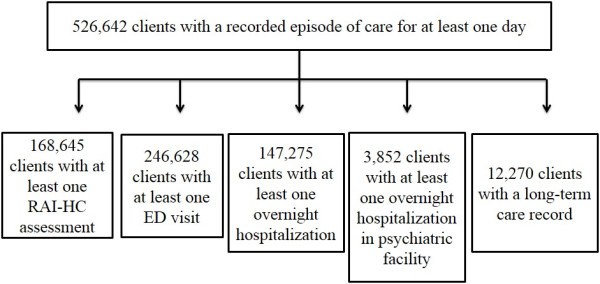
Number of home care clients with at least one home care episode in 2008-2009 with linkage to RAI-HC assessment, emergency department record, hospitalization, psychiatric inpatient admission, or long-term care home admission.

De-identified client-level data were obtained from the Canadian Institute for Health Information (CIHI) through linkable data cuts. At CIHI, the encrypted health card number, the province issuing the health card number, the birth year, and birth month were used to do the linkage. The data were prepared by identifying HC clients in Ontario where the data from recent years were available. The health card numbers were then used to identify health-service records in other available datasets (DAD, NACRS, CCRS, MHRS) for the years during and around the time of an identified HC episode. All assembled records then had a common encryption algorithm applied to the health card numbers to enable the researchers to establish person-level linkage without the release of any real-world identifiers. The number of HC clients identified by this linkage of various databases is summarized in Figure 1.

*The Determination of an adverse event* involved case screening based on previous literature [2-4,9] and was further developed in our research. The population at risk was operationally defined as HC clients who were in a HC program during the reporting period either with or without a RAI-HC assessment, depending on the availability of data and the type of events. The at-risk clients were followed forward from their case-open date until an event was identified in one of the acute-care data sets. The case-screening period covered 30 days after the client’s discharge from the HC program. Case screening was conducted by two teams of researchers consisting of statisticians, clinicians, and health-service researchers. They worked independently but met weekly to review case-screening criteria, to resolve operational definition issues, and to establish Statistical Analysis Software (SAS) coding for each adverse event.

*The Incidence rate* was determined by calculating the number of clients with an adverse event recorded in the DAD/NACRS/MHRS data divided by the number of clients who were in the HC program during the calendar year. Some clients may have had multiple adverse events of the same type (e.g. multiple injurious falls) or multiple events of different types. Multiple occurrences of the same event during the reporting period were only counted once. We examined two specific consequences of adverse events: LTC facility admission and client death. LTC admission was identified by the recorded date of entry from CCRS records. Death was identified by any record in NACRS, DAD, or HC episode data, of a discharge deceased within the episode or 30 days following it.

### Analysis

There were two incidence rate measures calculated for each adverse event: one, the percentage of clients who experienced a new adverse event per year; and two, the rate per 1,000 HC days. An overall incidence rate was calculated by dividing the number of clients with at least one adverse event of any type by the number of clients who were in the HC program during the calendar year. Logistic regression analysis was used to determine the association between the adverse events and LTC placement or client death. Adjustment for known and measured risk factors was performed, with backwards elimination to produce a parsimonious model with significant covariates.

## Results

### Characteristics of the general population of home care clients

For 2009 the average age of HC clients in Ontario was 68.3 years (standard deviation 18.5). In that year 59% were female. At the time of intake to HC service 42% of clients were classified as requiring acute care, 28% requiring maintenance, 23% rehabilitation, and 7% long-term support. The average number of months a client spent in the HC program during that year was 4.9 (SD 4.4).

### Adverse events

The rates of adverse events identified in NACRS/ DAD/ MHRS for Ontario HC clients are presented in Table 1 for 2008 and 2009. Injurious falls, injuries from other than falls, and medication-related events resulting in an ED visit or hospitalization were the most frequently occurring. Examples of medication-related events include accidental poisoning, an adverse effect at therapeutic dose, an overdose, and a haemorrhagic disorder due to circulating anticoagulants. Sepsis/bacteraemia and delirium were also ranked among the top five adverse events. Deep vein thrombosis, diabetic foot ulcers, pressure ulcers, pulmonary emboli, venous leg ulcers, and suicide were less frequently identified events. The overall incidence rate for all adverse events was 13% for 2008 and 2009. That rate expressed in clients per 1,000 client-days, was 0.858 in 2008 and 0.892 in 2009.

**Table 1:** Incidence rates of adverse events identified in NACRS/ DAD/ MHRS for Ontario home care clients in 2008 and 2009

**Adverse event**	% (n^*^)	**Clients per 1,000 client-days**
	2008 (N^**^ **= 380,962)**	**2008**
	**2009 (N = 387,885)**	**2009**
**Injurious fall**	4.93(18,784)	0.333
	5.05 (19,603)	0.339
**Injury other than fall**	4.14 (15,758)	0.279
	4.30 (16,666)	0.288
**Medication-related ED or hospitalization**	2.96 (4,802)	0.200
	3.13 (5,515)	0.210
**Sepsis / Bacteraemia**	1.26 (4,802)	0.085
	1.42 (5,515)	0.095
**Delirium**	0.94 (3,577)	0.063
	1.05 (4,085)	0.071
**Deep vein thrombosis**	0.74 (2,811)	0.050
	0.84 (3,249)	0.056
**Diabetic foot ulcer**	0.40 (1,513)	0.027
	0.39 (1,502)	0.026
**Pressure ulcer (stage 2+)**	0.12 (437)	0.019
	0.12 (471)	0.020
**Pulmonary embolus**	0.28 (1,049)	0.008
	0.29 (1,144)	0.008
**Venus leg ulcer**	0.05 (203)	0.004
	0.06 (241)	0.004
**Suicide**^**+**^	--	--
	0.13 (503)	0.009
**Others**^**‡**^	0.43 (1,654)	0.029
	0.45 (1,763)	0.030
**Overall**	**12.72 (48,461)**	**0.858**
	**13.31 (51,631)**	**0.892**

^*^n = Number of home care clients with adverse event, i.e., the numerator of the incidence rate.

^**^N = Number of home care clients who are at risk of adverse event, i.e., the denominator of the incidence rate.

^+^Only 2009 rate is available because of very limited cases available for 2008.

^‡^ Other adverse events include wound infection, medical device-associated infections, new pressure ulcer less than stage 2+, new stasis ulcer or worsening, and any new injury.

Table 2 relates at-risk populations to specific adverse events. For example, only clients with an indwelling urethral catheter were considered at risk for a catheter-associated urinary tract infection (UTI), and only clients who had surgery were considered at risk for surgical site infection. Each of these events, along with medication-related incidents, also represents a sub-set of events that are more closely associated with specific healthcare interventions. We note here that catheter-associated UTI was the most frequent of the adverse events observed.

**Table 2:** Incidence rates of adverse events identified in NACRS/ DAD/ OMHRS for subgroups of Ontario home care clients in 2008 and 2009

**Adverse events in at risk sub-groups**	% (n*/ N**)	**clients per 1,000 client-days**
	**2008, 2009**	**2008, 2009**
**Surgical wound infection**		
(Surgical wound infection present on any ED visit or hospital admission within 30 days of a hospital discharge with open surgery but without infection recorded)	2.62 (1,286/49,086)	0.887
	2.81 (1,374/48,831)	0.954
**Ventilator-associated pneumonia**		
(Pneumonia present on any ED visit or hospital admission *within 30 days of RAI*-*HC assessment* among clients who had ventilator documented but didn’t have pneumonia recorded at the time of assessment)	1.68 (9/537)	0.562
	2.72 (15/552)	0.921
**Newly-detected catheter-associated UTI**		
(UTI present on any ED visit or hospital admission *within 30 days of RAI*-*HC assessment* among clients who had indwelling urinary catheter documented but didn’t have UTI recorded at the time of assessment)	8.22 (261/3,174)	2.866
	8.11 (243/2,997)	2.819
**Peripheral IV infection**		
(Bacteremia or localized skin infection present on any ED visit or hospital admission *within 60 days of RAI*-*HC assessment* among clients who had peripheral IV infusion documented at the time of assessment)	3.17 (45/1,421)	0.547
	2.76 (41/1,483)	0.475
**Central line IV infection**		
(Bacteremia or localized skin infection present on any ED visit or hospital admission *within 60 days of RAI*-*HC assessment* among clients who had central IV infusion documented at the time of assessment)	2.79 (59/2,118)	0.476
	3.95 (93/2,352)	0.679

* n = Number of home care clients with adverse event, i.e., the numerator of the incidence rate.

** N = Number of home care clients at risk of adverse event, i.e. the denominator of the incidence rate.

### Consequences of adverse events

Table 3 presents the results of our analysis of associations between adverse events and LTC facility placement. We accounted for client characteristics such as age, gender, dementia, pneumonia diagnosis, and priority for long-term-care placement using the MAPLe score. The MAPLe score is the Method for Assigning Priority Levels (MAPLe) algorithm for LTC facility placement, using data based on the RAI-HC [15]. We determined that an injurious fall was associated with increased odds of a LTC placement, whereas sepsis was associated with reduced odds of a LTC placement.

**Table 3:** Adjusted odds ratio estimates for long-term care facility placement among Ontario HC clients with at least one RAI-HC assessment

**Variable**		**Adjusted odds ratio**	**95% confidence interval**
**Age (years)**	65-74 vs <65	1.82	1.49, 2.23
	75-84 vs <65	2.52	2.11, 3.02
	85+ vs < 65	3.31	2.76, 3.95
**Female**		1.14	1.04, 1.24
**MAPLe score**^**‡**^	2 vs 1	1.45	1.16, 1.81
	3 vs 1	2.82	2.38, 3.33
	4 vs 1	3.42	2.88, 4.06
	5 vs 1	4.86	4.02, 5.88
**Dementia**		1.79	1.63, 1.98
**Injurious fall**		1.31	1.15, 1.49
**Sepsis**		0.43	0.26, 0.72
**Pneumonia**		0.70	0.65, 0.97

p-value = 0.4531 for goodness-of-fit test.

^‡^The MAPLe score is the Method for Assigning Priority Levels (MAPLe) algorithm for LTC home placement, using data based on the RAI-HC [15].

Several adverse events were associated with increased odds of death; specifically, injurious fall, medication-related incidents, sepsis, and delirium (Table 4).

**Table 4:** Adjusted odds ratio estimates for death among Ontario HC clients with at least one RAI-HC assessment

**Variable**		**Adjusted odds ratio**	**95% confidence interval**
**Age (years**)	65-74 vs < 65	1.34	1.20, 1.50
	75-84 vs < 65	1.24	1.12, 1.37
	85+ vs < 65	1.66	1.50, 1.84
**Female**		0.60	0.56, 0.64
CHESS score^*^	1 vs 0	1.12	1.02, 1.23
	2 vs 0	1.63	1.48, 1.79
	3 vs 0	2.15	1.93, 2.38
	4 vs 0	2.21	1.81, 2.69
	5 vs 0	11.23	1.30, 54.86
**Dementia**		0.90	0.83,0.98
**Injurious fall**		1.27	1.15, 1.41
**Medication related**		1.29	1.07, 1.55
**Delirium**		1.95	1.60, 2.37
**Sepsis**		4.31	3.70, 5.02
**Pneumonia**		2.65	2.41, 2.92

p-value < .002 for goodness-of-fit test.

^*^The CHESS score is Changes in Health, End Stage Disease, Signs and Symptoms [16].

## Discussion

Our investigation determined that the overall incidence rate of adverse events for publically-funded Ontario HC clients was 13% for 2008 and 2009. This rate confirms the findings of two previous studies [2,4]. The enhanced focus of our study was on adverse events among those HC clients that were associated with an ED visit or hospitalization. The two previous studies had identified events through HC clients’ charts [2] or events recorded in standardized assessment [4]. In spite of the different methodologies used, the incidence rates were consistent.

Our study was able to provide more detailed and helpful information related to different types of adverse events. Injurious falls, injuries from other than falls, and medication-related adverse events were the most frequent types of events observed.

Approximately 5% of the Ontario HC clients studied had falls that resulted in injuries requiring an ED or hospital visit. That incidence rate is in the low range of the previously reported 5% to 25% of falls that result in injury [17]. Approximately, one in three Canadians aged 65 and older will have an injurious fall each year. That is a total of 1.3 million seniors experiencing a fall [18]. Unintentional falls will account for 84% of all hospitalization due to injury [19], at a cost of approximately $2.9 billion annually [20]. Of those hospitalizations 23.7% to 36.8% will result in death in the hospital [21]. This safety issue has staggering implications for healthcare services and costs. As the population ages the number of older adults receiving HC will continue to increase. These findings emphasize the need to develop effective policies and strategies that will target falls prevention as a strategy in promoting safer care and managing cost.

For the most part, there was no way to determine from the secondary data available whether the events observed were due to the ‘*plans or actions taken during the provision of health care*’ or due to an underlying disease, or injury, or other causes. However, there was a subset of events identified that was more closely associated with specific care interventions such as medication- related ED visits or hospitalization, catheter associated UTI, central and peripheral line infection, and surgical site infection.

New catheter-associated infection was identified as a relatively frequent adverse event occurring in about 8% of HC clients who had an indwelling urethral catheter inserted in the previous 30 days. The prevalence rate of catheter-associated UTIs in primary and community health care was reported to be 8-10% in one study [22] and 21% in another study of frail women living in the community in Italy [23]. The mean infection rate for symptomatic UTI among patients with urethral catheter in four US home health agencies was 4.5 per 1,000 device-days (range 2.7-6.2) [24] and 2.8 per 1,000 device-days in another US study [25]. The rate per 1,000 HC days in our study was 2.8%, which is within range of these other studies.

The incidence of medication-related events identified through ED and hospital visits was 3%. This rate is noticeably lower than the 12% rate reported in one prospective study of medication-related visits to the ED [26] but close to the 4.7% rate reported in another study [27]. There is evidence that medication-related events are often under-detected in emergency department settings [27], making it plausible that the rate observed in this study is conservative.

The consequences of adverse events that we analyzed were LTC facility admission and client death. There was no way to determine from the secondary data available whether the events observed in this study were preventable and thus it is not possible to determine the extent to which the consequences could have been avoided. It is noteworthy that an injurious fall, which was the most frequently occurring adverse event, was associated with increased odds of both LTC facility admission and client death. Even if nothing else were addressed, we could significantly improve outcomes for some HC clients by reducing the risk and incidence of injurious falls. While Sepsis was a relatively infrequent adverse event, when it occurred it was associated with a significant increase in the risk of death. Delirium was associated with a twofold increase in the odds of death. By corollary, sepsis was associated with reduced odds of LTC facility admission, likely because the clients who experienced this outcome were more likely to be hospitalized.

### Strength and limitations

Our study engaged a large population of HC clients in Ontario, where well-established secondary health databases and the RAI-HC instrument, a highly reliable and validated assessment tool [13,14] were available for research support. Although there are other published studies that address HC safety, to our knowledge this is the first one that has investigated adverse events associated with ED visits or hospitalization. This provides a valuable dimension to the examination of safety concerns recorded in HC contexts.

The analysis of adverse event data is challenging work. Some types of events are particularly difficult to interpret accurately because of unique factors that include: non-recognition or non-reporting of medication errors that present in the ED [23]; falls that do not leave visible marks or for which hospital attention is not sought; pressure ulcers that require personal examination; or sub-clinical infections that are likely to be under-reported both through RAI-HC assessment and by encounters with the ED or hospital. As noted above there is generally no reliable way to determine with certainty, from the secondary data, whether the events/outcomes observed in the study were due to the care delivered in the home or to an underlying disease, patient behaviour, injury, or other causes.

## Conclusions

The overall incidence rate of adverse events for Ontario HC clients in 2008 and 2009 was approximately 13%. That rate confirms rates previously reported in Canada and the United States [2,4]. It is a rate that has significant implications for the delivery and costing of HC services. This study has highlighted the importance of safety events in the HC setting and has identified associations between adverse events and adverse outcomes that will guide the establishing of priority areas for intervention.

It was not always possible to determine the locus of responsibility from the secondary databases, thus we could not determine the extent to which adverse events observed in our study were due to human error or system failure. However, it is noteworthy that many of the types of events observed, such as medication errors and falls, are potentially preventable. Strategies designed to improve the safety of HC clients need to focus on reducing the risk of falls and other injuries in the home, improving medication management, and promoting recognition of early signs and symptoms of sepsis/bacteraemia and delirium followed by prompt intervention. Policies are needed to improve the system of care by improving the assessment and monitoring of risk, education, and by improving care coordination and communication [8]. Tools already exist in Canada that could be used to assess and manage clients at risk for falls or other adverse events. The interRAI clinical assessment protocols [28] and the Registered Nurses’ Association of Ontario (RNAO) best-practice guidelines [29,30] are two examples. Implementation of the full clinical capabilities of the RAI-HC in Canada should be a priority. Advancement of electronic documentation is another initiative that will support improvement by facilitating access to information, enabling more timely communication, and supporting the standardization of care processes. At the strategic level it will be important to work with health jurisdictions to effect changes in accreditation standards, safety monitoring, and funding policies to ensure that the safety recommendations are implemented in practice.

## Abbreviations

AE: Adverse Event; CCRS: Continuing Care Reporting System; CHESS: Changes in Health, End Stage Disease, Signs and Symptoms; CHF: Congestive Heart Failure; CI: Confidence Interval; CIHI: Canadian Institute of Health Information; COPD: Chronic Obstructive Pulmonary Disease; DAD: Discharge Abstract Database; DSM: Diagnostic and Statistics Manual of Mental Disorders; ED: Emergency Department; HC: Home Care; HCRS: Home Care Reporting System; HI: Harmful Incident; IV: Intravenous; LTC: Long-Term Care; MAPLe: Method for Assigning Priority Levels; MHRS: Mental Health Reporting System; MRDx: Most responsible diagnosis; NACRS: National Ambulatory Care Reporting System; OMHRS: Ontario Mental Health Reporting System; ON: Ontario; RAI-HC: Resident Assessment Instrument-Home Care; RAI-MH: Resident Assessment Instrument-Mental Health; UTI: Urinary Tract Infection; WHO: World Health Organization.

## Competing interests

The authors have no competing interests to declare.

## Authors’ contributions

DMD was the principle investigator, and was responsible for leading the development of the proposal for funding, guiding data analysis, and drafted the paper for publication. JPH participated in development of the funding proposal, co-lead a sub-project and reviewed the paper for publication. RB co-lead the proposal for funding, was co-principal investigator of the research, provided input into data analysis, and contributed to the paper for publication. GRB participated in development of the funding proposal, provided input into the data analysis, and reviewed the paper for publication. JP and XL co-lead the data analysis and contributed to the development of the paper for publication. DD, AG, GH, HL, LM, NW, and MO contributed to development of the funding proposal, contributed to data analysis, reviewed and provided input into the draft publication. AF, GQ, SN, MJ contributed to data analysis and development of the paper for publication. OY and LD contributed to preparation of the paper for publication. All authors have read and approved the final manuscript.

## Authors’ information

DMD holds a doctorate degree with expertise in outcomes research and patient safety and is now professor emeritus at the Lawrence S. Bloomberg Faculty of Nursing, University of Toronto. JPH is a health services researcher and Ontario Home Care Research and Knowledge Exchange Chair. RB has a doctorate in psychology and has expertise in optimal use, access, quality, and safety of health services. GRB has expertise in patient safety and a doctorate degree in sociology. JWP is a health services researcher with a doctorate in health studies.

XL is a statistician and research associate at University of Toronto. DD is a nurse and Director of Monitoring and Evaluation for the Continuing Care Branch of the Nova Scotia Department of Health. AG is a scientist at Women’s College Research Institute in Toronto. GH is a physician, Schlegel Research Chair for Geriatric Medicine and Associate Professor, Research Institute for Aging and School of Public Health and Health Systems, University of Waterloo.

HL is a nurse and Director, Program Advancement at Saint Elizabeth Health Care. LM is a researcher with the Home Care Program, Winnipeg Regional Health Authority. MO is a physician specializing in family medicine with research expertise in patient safety in community based practices. NW is past manager of the Home and Continuing Care program at the Canadian Institute for Health Information. LD is Chief Care Innovations Officer, Ontario Association of Community Care Access Centres. ADF is a post-doctoral fellow specializing in aging. GQ is a statistician. SMN is a physiotherapist with a doctorate in epidemiology. QY is a research assistant at University of Toronto. CM is a nurse with expertise in wound management. MJ is a data manager at the University of Waterloo.

## Pre-publication history

The pre-publication history for this paper can be accessed here:

http://www.biomedcentral.com/1472-6963/13/227/prepub

## Supplementary Material

Additional files 1 Adverse events and data sources for incidence rate calculation. Click here for file
