# Supplementary material for: Adverse events among Ontario home care clients associated with emergency room visit or hospitalization: a retrospective cohort study
Source: BMC Health Serv Res. 2013 Jun 22;13:227. doi: 10.1186/1472-6963-13-227 (PMC3751652; doi:10.1186/1472-6963-13-227)
Supplement: Additional files 1 — Adverse events and data sources for incidence rate calculation. [file 1472-6963-13-227-S1.doc]

**Adverse Events and Data Sources for Incidence Rate Calculation**

| **Adverse events** | **Technical definition** | **Data sources** |
| --- | --- | --- |
| Injurious fall presenting to ED or hospitalization | Denominator: Number of non-end-of-life home care clients with a recorded episode of care for at least 1 day during the calendar year.  Numerator: Number of non-end-of-life home care clients who had a fall-related injury recorded for any unplanned and unscheduled ED visit, or recorded as a pre-admit condition for any overnight hospitalization.  Inclusions/Exclusions: The date of registration in ED or admission to a hospital should be within a home care episode including the 30-day extension after discharge. A fall code (W0 or W1) must be present to be considered an injurious fall. ED visits and hospitalizations with a date of registration or admission that coincided with the date of case open were excluded | ICD-10 codes: S00-S09, S10-S19, S20-S29, S30-S-39, S40-S49, S50-S59, S60-S69, S70-S79, S80-S89, S90-S99, T00-T07, T08-T14, W0, W1 |
| Injury other than fall presenting to ED or hospitalization | Denominator: Number of non-end-of-life home care clients with a recorded episode of care for at least 1 day during the calendar year.  Numerator: Number of non-end-of-life home care clients who had a non-fall-related injury recorded for any unplanned and unscheduled ED visit, or recorded as a pre-admit condition for any overnight hospitalization.  Inclusions/Exclusions: The date of registration in ED or admission to a hospital should be within a home care episode, including the 30-day extension after discharge. ED visits and hospitalizations with a date of registration or admission that coincided with the date of case open were excluded. | ICD-10 codes: S00-S09, S10-S19, S20-S29, S30-S-39, S40-S49, S50-S59, S60-S69, S70-S79, S80-S89, S90-S99, T00-T07, T08-T14, T20-T32, W20-W49, W50-W64, W65-W74, W65-W74, W75-W84, W85-W99, X00-X09 |
| Medication-related event presenting to ED or hospitalization | Denominator: Number of non-end-of-life home care clients with a recorded episode of care for at least 1 day during the calendar year.  Numerator: Number of non-end-of-life home care clients who had any medication-related event recorded for any unplanned and unscheduled ED visit, or recorded as a pre-admit condition for any overnight hospitalization.  Inclusions/Exclusions: The date of registration in ED or admission to a hospital should be within a home care episode, including the 30-day extension after discharge. ED visits and hospitalizations with a date of registration or admission that coincided with the date of case open were excluded. | ICD-10 codes: T3, T4, Y4, Y5, X40, X41, X42, X43, X44, T50, E15,  E10.0, E10.1, E11.0, E11.1, E13.0, E13.1, E14.0, E14.1, L27.0, L27.1, L23.3, L24.4, L25.1, E16.0, D68.3, I95.1, R50.2, D52.1, D59.0, D59.2, D61.1, E03.2, E06.4, E23.1, E24.2, E27.3, E66.1, G21.0, G21.1, G24.0, G25.1, G25.4, G44.4, G62.0, G72.0, H26.3, H35.8, H40.6, K85.3, L10.5, L43.2, M10.2, M32.0, M80.4, M81.4, M83.5, M87.1 |
| Sepsis / Bacteraemia presenting to ED or hospitalization | Denominator: Number of non-end-of-life home care clients with a recorded episode of care for at least 1 day during the calendar year  Numerator: Number of non-end-of-life home care clients who had sepsis/bacteraemia recorded in any unplanned and unscheduled ED visit, or recorded as a pre-admit condition for any overnight hospitalization  Inclusions/Exclusions: The date of registration in ED or admission to a hospital should be within a home care episode, including the 30-day extension after discharge. ED visits and hospitalizations with a date of registration or admission that coincided with the date of case open were excluded. | ICD-10 codes: A40, A41, O85, A02.1, A22.7, A26.7, A32.7, A39.4, A42.7, A49.9, B00.7, B37.7, R57.2, R65.1, T88.0, J95.01, K91.41, K91.44, K91.61, N99.51 |
| Delirium presenting to ED or hospitalization | Denominator: Number of non-end-of-life home care clients with at least one day of service during the year  Numerator: Number of non-end-of-life home care clients with delirium present on any unplanned and unscheduled ED visit, or recorded as a pre-admit condition for any overnight hospitalization in a general hospital, or with delirium recorded on the initial RAI-MH assessment for any psychiatric hospital admission.  Inclusions/Exclusions: The date of registration in ED or admission to a hospital should be within a home care episode, including the 30-day extension after discharge. Admissions to psychiatric hospitals not related to mental health problems were not included. Only the initial RAI-MH assessment was used to search for the presence of delirium. ED visits and hospitalizations with a date of registration or admission that coincided with the date of case open were excluded. | ICD-10 code: F05  RAI-MH items:  f3a=2 or f3b=2 or f3c=2 or f3d=2 or f3e=2 or f3f=2 |
| Deep vein thrombosis presenting to ED or hospitalization | Denominator: Number of non-end-of-life home care clients with a recorded episode of care for at least 1 day during the calendar year.  Numerator: Number of non-end-of-life home care clients who had Deep Vein Thrombosis recorded for any unplanned and unscheduled ED visit, or recorded as a pre-admit condition for any overnight hospitalization.  Inclusions/Exclusions: The date of registration in ED or admission to a hospital should be within a home care episode, including the 30-day extension after discharge. ED visits and hospitalizations with a date of registration or admission that coincided with the date of case open were excluded. | ICD-10 code:  I80.2 |
| Diabetic Foot ulcer presenting to ED or hospitalization | Denominator: Number of non-end-of-life home care clients with a recorded episode of care for at least 1 day during the calendar year.  Numerator: Number of non-end-of-life home care clients who had a diabetic foot ulcer present as the main problem for any unplanned and unscheduled ED visit, or as the main diagnosis (not a post-admission condition) recorded for any overnight hospitalization.  Inclusions/Exclusions: The date of registration in ED or admission to a hospital should be within a home care episode, including the 30-day extension after discharge. According to the 2012 Canadian Coding Standards, if a post-admit comorbidity qualifies as the most responsible diagnosis (MRDx), it must be recorded as both the MRDx and as a Diagnosis Type=2. So, if the main diagnosis is a diabetic foot ulcer code and the same code appears as a Type 2 secondary diagnosis, the main diagnosis is considered to be a post-admit condition and is therefore removed from the numerator. ED visits and hospitalizations with a date of registration or admission that coincided with the date of case open were excluded. | ICD-10 code:  E10.70, E10.71, E11.70, E11.71, E13.70, E13.71, E14.70, E14.71  For Winnipeg, the ICD-10 codes are:  E10.7, E11.7, E13.7, E14.7 |
| Pressure ulcer (stage 2+) presenting to ED or hospitalization | Denominator: Number of non-end-of-life home care clients with a recorded episode of care for at least 1 day during the calendar year.  Numerator: Number of non-end-of-life home care clients who had a pressure ulcer (stage 2, 3 or 4) present as the main problem for any unplanned and unscheduled ED visit, or as the main diagnosis (not a post-admission condition) recorded for any overnight hospitalization.  Inclusions/Exclusions: The date of registration in ED or admission to a hospital should be within a home care episode, including the 30-day extension after discharge. According to the 2012 Canadian Coding Standards, if a post-admit comorbidity qualifies as the most responsible diagnosis (MRDx), it must be recorded as both the MRDx and as a Diagnosis Type=2. So, if the main diagnosis is a pressure ulcer code, and the same code appears as the secondary diagnosis with a Type=2, the main diagnosis is considered to be a post-admit condition and is therefore removed from the numerator. ED visits and hospitalizations with a date of registration or admission that coincided with the date of case open were excluded. | ICD-10 codes: L89.1, L89.2, L89.3, L89.4, L89.5, L89.8, L89.9 |
| Pulmonary embolus presenting to ED or hospitalization | Denominator: Number of non-end-of-life home care clients with a recorded episode of care for at least 1 day during the calendar year.  Numerator: Number of non-end-of-life home care clients who had pulmonary embolus present as the main problem for any unplanned and unscheduled ED visit, or as the main diagnosis (not a post-admission condition) recorded for any overnight hospitalization.  Inclusions/Exclusions: The date of registration in ED or admission to a hospital should be within a home care episode including the 30-day extension after discharge. According to the 2012 Canadian Coding Standards, if a post-admit comorbidity qualifies as the most responsible diagnosis (MRDx), it must be recorded as both the MRDx and as a Diagnosis Type=2. If the main diagnosis is the pulmonary embolus code, and the same code appears as the secondary diagnosis with a Type=2, the main diagnosis is considered to be a post-admit condition and is therefore removed from the numerator. ED visits and hospitalizations with a date of registration or admission that coincided with the date of case open were excluded. | ICD-10 codes: I26 |
| Venous leg ulcer presenting to ED or hospitalization | Denominator: Number of non-end-of-life home care clients with a recorded episode of care for at least 1 day during the calendar year.  Numerator: Number of non-end-of-life home care clients who had venous leg ulcer present as the main problem for any unplanned and unscheduled ED visit, or as the main diagnosis (not a post-admission condition) recorded for any overnight hospitalization.  Inclusions/Exclusions: The date of registration in ED or admission to a hospital should be within a home care episode, including the 30-day extension after discharge. According to the 2012 Canadian Coding Standards, if a post-admit comorbidity qualifies as the most responsible diagnosis (MRDx), it must be recorded as both the MRDx and as a Diagnosis Type=2. If the main diagnosis is a venous leg ulcer code, and the same code appears as the secondary diagnosis with a Type=2, the main diagnosis is considered to be a post-admit condition and is therefore removed from the numerator. ED visits and hospitalizations with a date of registration or admission that coincided with the date of case open were excluded. | ICD-10 codes: I83.0, I83.2 |
| Suicide/suicide attempt presenting to ED | Denominator: Number of non-end-of-life home care clients with a recorded episode of care for at least 1 day during the calendar year.  Numerator: Number of non-end-of-life home care clients who had intentional self-harm or intentional self-poisoning recorded for any unplanned and unscheduled ED visit, or recorded for any overnight hospitalization in a general hospital, or had self-injurious attempt within last 7 days with intent to kill him/herself recorded on the initial RAI-MH assessment for any psychiatric hospital admission.  Inclusions/Exclusions: The date of registration in ED or admission to a hospital should be within a home care episode, including the 30-day extension after discharge. Admissions to psychiatric hospitals that were not related to mental health problems were not included. ED visits and hospitalizations with a date of registration or admission that coincided with the date of case open were excluded. | ICD-10 codes: X6, X7, X80, X81, X82, X83, X84, X79, X80, X81, X82, X83, X84  RAI-MH items:  d1a in (4 5) and d1b =1 |

| **Others – Subgroup of all home care clients with His identified in NACRS/DAD** | | |
| --- | --- | --- |
| Surgical wound infection  (within 30 days of hospital surgery without infection) | Denominator: Number of non-end-of-life home care clients with a recorded episode of care for at least 1 day during the calendar year who received surgery as main intervention, but without surgical wound infection recorded on discharge from a general hospital.  Numerator: Number of non-end-of-life home care clients who had a surgical wound infection recorded for any unplanned and unscheduled ED visit, or recorded as a pre-admit condition for any overnight hospitalization, within 30 days of a hospital discharge with surgery.  Inclusion/exclusions: Surgery done within 29 days prior to the home care ‘case open date’ is included and the surgery should be done at least one day before discharge from home care program. The calendar year of the hospital discharge date, with surgery performed during the hospitalization, was used to assign the clients to each year for the yearly rate calculation. The date of registration in ED, or admission to a hospital with surgical wound infection recorded, should be within a home care episode, including the 30-day extension after discharge. ED visits and hospitalizations with a date of registration or admission that coincided with the case open date and also had a documented surgical wound infection were excluded. | The surgery is defined by the ICD-10 major surgery CMG groups. The CMG codes are: 1 2 4 5 6 7 8 9 12 71 72 110 111 112 113 114 118 160 162 163 165 166 167 168 169 170 171 172 181 182 220 221 222 223 224 225 270 271 272 273 274 300 302 303 305 312 313 315 316 317 318 319 320 321 326 420 421 422 450 451 452 453 454 462 500 501 503 504 611 612 710 725 726 727 729 730 731 732 733 735.  The surgical wound infection ICD-10 codes: T81.4, O86.0 |

| **Others – RAI-HC clients with HI identified in NACRS/DAD** | | |
| --- | --- | --- |
| Ventilator-associated Pneumonia  (Pneumonia present on any ED visit or hospital admission *within 30 days of RAI-HC assessment* among home care clients who had ventilator documented and didn’t have pneumonia recorded at the time of assessment) | Denominator: Number of non-end-of-life home care clients with a ventilator but without pneumonia documented at the time of RAI-HC assessment  Numerator: Number of non-end-of-life home care clients with pneumonia recorded on any ED visit, or as a pre-admit condition recorded for an overnight hospitalization, within 30 days after RAI-HC assessment with ventilator documented but without pneumonia recorded at the time of RAI-HC assessment  Inclusion/Exclusions: The RAI-HC assessment should be within a home care episode. RAI-HC assessments were excluded if the end-of-life status was identified at the time of the assessment. RAI-HC assessments that were done during a hospitalization were excluded. The calendar year of the RAI-HC assessment with ventilator documented was used to assign the clients to each year for the yearly rate calculation. The date of registration in ED or admission to a hospital should be within a home care episode, including the 30-day extension after discharge. | ICD-10 codes to define pneumonia: A06.5, A20.2, A22.1, A48.1, A70, B01.2, B05.2, B25.0, B37.1, B44.1, B45.0, B58.3, B59, J10.0, J11.0, J12.0, J12.1, J12.2, J12.8, J13, J14, J15, J15.0, J15.1, J15.2, J15.3, J15.4, J15.5, J15.6, J15.7, J15.8, J15.9, J16.0, J16.8, J17.0, J17.1, J17.2, J17.3, J17.8, J18.0, J18.1, J18.2, J18.8, J18.9, J69.0, J85.1.  The RAI-HC items to define the denominator:  k8e (not equal 1) and p2b in (1, 2) and j1u =0 |
| Newly-detected Catheter-associated UTI  (UTI present on any ED visit or hospital admission *within 30 days of RAI-HC assessment* among clients who had indwelling urinary catheter documented and didn’t have UTI recorded at the time of assessment) | Denominator: Number of non-end-of-life home care clients with an indwelling urinary catheter documented but without UTI recorded at the time of RAI-HC assessment  Numerator: Number of non-end-of-life home care clients with UTI recorded on any ED visit, or as a pre-admit condition recorded for an overnight hospitalization, within 30 days after RAI-HC assessment with indwelling urinary catheter documented but without UTI recorded at the time of assessment.  Inclusion/Exclusions: The RAI-HC assessment should be within a home care episode. RAI-HC assessments were excluded if the end-of-life status was identified at the time of the assessment. RAI-HC assessments done during a hospitalization were excluded. The calendar year of the RAI-HC assessment with indwelling urinary catheter documented was used to assign the clients to each year for the yearly rate calculation. The date of registration in ED or admission to a hospital should be within a home care episode, including the 30-day extension after discharge. | ICD-10 codes to define UTI: N10, N12, N16, N15.1, N30.0, N34.0, N34.1, N34.2 , N39.0, N28.83, N28.84, N28.85, B37.4  The RAI-HC items to define the denominator:  k8e (not equal 1) and i2b=1 and i1a in (1 2 3 4 5) and j1w=0 |
| Peripheral IV Infection  (a bacteremia or localized skin infection present on any ED visit or hospital admission *within 60 days of RAI-HC assessment,* among home care clients who had IV infusion -peripheral documented at the time of assessment) | Denominator: Number of non-end-of-life home care clients with IV infusion - peripheral documented at the time of RAI-HCassessment  Numerator: Number of non-end-of-life home care clients with a sepsis/bacteremia or localized limb skin infection recorded on any ED visit, or as a pre-admit condition recorded for an overnight hospitalization, within 60 days after RAI-HC assessment with IV infusion - peripheral documented at the time of RAI-HC assessment  Inclusion/Exclusions: The RAI-HC assessment should be within a home care episode. RAI-HC assessments were excluded if the end-of-life status was identified at the time the assessment. RAI-HC assessments done during a hospitalization were excluded. The calendar year of the RAI-HC assessment with IV infusion - peripheral documented was used to assign the clients to each year for the yearly rate calculation. The date of registration in ED or admission to a hospital should be within a home care episode including the 30-day extension after discharge. | ICD-10 codes to define a bacteremia or localized skin:  A40, A41, O85, A02.1, A22.7, A26.7, A32.7, A39.4, A42.7, A49.9, B00.7, B37.7, R57.2, R65.1, T88.0, L08.8, L08.9, J95.01, K91.41, K91.44, K91.61, N99.51, L03.10  The RAI-HC items to define the denominator:  k8e (not equal 1) and p2i in (1 2) |
| Central line IV Infection  (a bacteremia or localized skin infection present on any ED visit or hospital admission *within 60 days of RAI-HC assessment* among clients who had IV infusion - central documented at the time of assessment) | Denominator: Number of non-end-of-life home care clients with IV infusion - central documented at the time of RAI-HCassessment  Numerator: Number of non-end-of-life home care clients with a sepsis/bacteremia or localized trunk skin infection recorded on any ED visit, or as a pre-admit condition recorded for an overnight hospitalization, within 60 days after RAI-HC assessment with IV infusion - central documented at the time of RAI-HC assessment  Inclusion/Exclusions: The RAI-HC assessment should be within a home care episode. RAI-HC assessments were excluded if the end-of-life status was identified at the time the assessment. RAI-HC assessments done during a hospitalization were excluded. The calendar year of the RAI-HC assessment with IV infusion - central documented was used to assign the clients to each year for the yearly rate calculation. The date of registration in ED or admission to a hospital should be within a home care episode including the 30-day extension after discharge. | The ICD-10 codes to define a bacteremia or localized skin infection:  A40, A41, O85, A02.1, A22.7, A26.7, A32.7, A39.4, A42.7, A49.9, B00.7, B37.7, R57.2, R65.1, T88.0, L08.8, L08.9, J95.01, K91.41, K91.44, K91.61, N99.51, L03.30  The RAI-HC items to define the denominator:  k8e (not equal 1) and p2h in (1 2) |
| Any wound infection or complicated wound  (surgical wound infection or complicated wound present on any ED visit or hospital admission *within 365 days of RAI-HC assessment* among clients who had wound treatment or ulcer documented at the time of assessment) | Denominator: Number of non-end-of-life home care clients with a documented surgical wound or ulcer or any skin problems requiring treatment, or receiving wound/ulcer care or skin treatment at the time of RAI-HCassessment  Numerator: Number of non-end-of-life home care clients with surgical wound infection or complicated wound recorded on any unplanned and unscheduled ED visit, or as a pre-admit condition recorded for an overnight hospitalization, within 365 days after RAI-HC assessment with documented surgical wound or ulcer or any skin problems requiring treatment or with wound/ulcer care or skin treatment recorded at the time of the RAI-HC assessment.  Inclusion/Exclusions: The RAI-HC assessment should be within a home care episode. RAI-HC assessments were excluded if the end of life status was identified at the time the assessment. RAI-HC assessments done during a hospitalization were excluded. The calendar year of the RAI-HC assessment with a documented surgical wound or ulcer or any skin problems requiring treatment or with wound/ulcer care or skin treatment documented was used to assign the clients to each year for the yearly rate calculation. The date of registration in ED or admission to a hospital should be within home care episode, including the 30-day extension after discharge. | ICD-10 codes for any wound infection or complicated wound:  T81.4, O86.0 J95.01, S01.01, S01.11, S01.21, S01.31, S01.41, S01.51, S01.71, S01.81, S01.91, S11.01, S11.11, S11.21, S11.71, S11.81, S11.91, S21.01, S21.11, S21.21, S21.71, S21.81, S21.91, S41.01, S41.11, S41.71, S41.81, S51.01, S51.71, S51.81, S51.91, S61.01, S61.11, S61.71, S61.81, S61.91, S71.01, S71.11, S71.71, S71.81, S81.01, S81.71, S81.81, S81.91, S91.01, S91.11, S91.31, S91.71, T01.01, T01.11, T01.21, T01.31, T01.61, T01.81, T01.91  S31.001, S31.101, S31.111, S31.121, S31.131, S31.191, S31.201, S31.301, S31.401, S31.501, S31.701, S31.801 |
| Any wound infection or complicated wound (continued) |  | The RAI-HC items to define the denominator: k8e (not equal 1) and (n2a>1 or n2b>1 or n3a=1 or n3b=1 or n3c=1 or n3d=1 or n5a=1 or n5b=1 or n5c=1 or n5d=1 or p2y>0) |
| **Others –RAI-HC informed only** | | |
| New or worsening pressure ulcer | Denominator: Number of non-end-of-life home care clients with at least one follow-up assessment (T2) during the year, and no pressure ulcer or a pressure ulcer less than Stage 4 at the prior assessment (T1).  Numerator: Number of non-end-of-life home care clients who developed a new stage 2+ pressure ulcer at follow-up assessment (T2), or had a more advanced pressure ulcer than the baseline assessment (T1).  Inclusions/Exclusions. Clients with two consecutive RAI-HC assessments (T1, T2) must belong to the same HC episode. Clients with a Stage 4 pressure ulcer at baseline assessment (T1) were excluded because Stage 4 is the highest Stage that an ulcer can be coded and are not eligible for worsening.  Clients were excluded if the period between the two consecutive assessments was less than 7 days or greater than 15 months. The calendar year of the T2 assessment was used to assign the pairs to each year for the yearly rate calculation. Clients with pairs of RAI-HC assessments were excluded if end-of-life status was identified at T1 or T2, or if the T2 assessment was done during a hospitalization. | RAI-HC item to define new pressure ulcer or stage worsening: (n2a_t2>=2 and n2a_t1<2) or (n2a_t2>n2a_t1 and n2a_t1>=2)  n2a - presence of pressure ulcer anywhere on the body [Code 0 if no ulcer, otherwise  record the highest ulcer stage  (Stage 1–4)]. |
| New or worsening stasis ulcer | Denominator: Number of non-end-of-life home care clients with at least one follow-up assessment (T2) during the year, and no stasis ulcer or a stasis ulcer in a stage less than 4 at the prior assessment (T1).  Numerator: Number of non-end-of-life home care clients who developed a new stage 2+ stasis ulcer at follow-up assessment (T2) or had a more advanced stasis ulcer than the baseline assessment (T1).  Inclusions/Exclusions. Clients with two consecutive RAI-HC assessments (T1, T2) must belong to the same HC episode. Clients with a Stage 4 stasis ulcer at baseline assessment (T1) were excluded because Stage 4 is the highest Stage that an ulcer can be coded and is not eligible for worsening.  Clients were excluded if the period between the two consecutive assessments was less than 7 days or greater than 15 months. The calendar year of the T2 assessment was used to assign the pairs to each year for the yearly rate calculation. Clients with pairs of RAI-HC assessments were excluded if end-of-life status was identified at T1 or T2, or if the T2 assessment was done during a hospitalization. | RAI-HC items to define new stasis ulcer or worsening: (n2b_t2>=2 and n2b_t1<2) or (n2b_t2>n2b_t1 and n2b_t1>=2).  n2b - presence of stasis ulcer anywhere on the body [Code 0 if no ulcer, otherwise  record the highest ulcer stage  (Stage 1–4)]. |
| Any new injury | Denominator: Number of non-end-of-life home care clients with at least one follow-up assessment during the year, with no injury recorded at the baseline (T1).  Numerator: Number of non-end-of-life home care clients with any of four types of injury (Hip fracture, other fractures, unexplained injuries/broken bones or burns, second or third degree Burns) recorded at the follow-up assessment (T2).  Inclusions/Exclusions. Clients having at least two consecutive RAI-HC assessments (T1, T2) were included if the period between the two consecutive assessments was at least 7 days but not greater than 15 months, and the two consecutive assessments should belong to the same home care episode. The calendar year of the T2 assessment was used to assign the pairs to each year for the yearly rate calculation. Pairs were excluded if end-of-life status was identified at T1 or T2, or if the T2 assessment was done during a hospitalization. | RAI-HC item to define any injury:  j1n in (1,2) or j1o in (1,2) or k9c=1 or n3a=1 |
| New Caregiver Distress | Denominator: Number of non-end-of-life home care clients with at least one follow-up assessment during the year with no caregiver distress recorded at the baseline (T1).  Numerator: Number of non-end-of-life home care clients with caregiver distress recorded at the follow-up assessment (T2).  Inclusions/Exclusions. Clients having at least two consecutive RAI-HC assessments (T1, T2) were included if the period between the two consecutive assessments was at least 7 days but not greater than 15 months, and the two consecutive assessments should belong to the same home care episode. The calendar year of the T2 assessment was used to assign the pairs to each year for the yearly rate calculation. Pairs were excluded if end-of-life status was identified at T1 or T2, or if the T2 assessment was done during a hospitalization. If there were no primary or secondary informal helpers at the time of either T1 or T2 RAI-HC assessment, then the assessment pair (T1 T2) was excluded. | RAI-HC item to define caregiver distress: (g1ea in (0 1) or g1eb in (0 1)) and (g2a=1 or g2c=1) |
